# It’s a long way to the top (if you want to personalize immunotherapy)

**DOI:** 10.1186/s40425-016-0207-0

**Published:** 2017-01-17

**Authors:** Sarah Haebe, Oliver Weigert

**Affiliations:** 1 Department of Medicine III, Laboratory for Experimental Leukemia and Lymphoma Research (ELLF), Ludwig-Maximilians-University, Max-Lebsche Platz 30, 81377 Munich, Germany; 2 German Cancer Consortium (DKTK) and German Cancer Research Center (DKFZ), Heidelberg, Germany

**Keywords:** Immunotherapy, Follicular lymphoma, Somatic mutation, Neoepitope

## Abstract

Harnessing the immune system to attack tumor cells by targeting tumor-associated or –preferably– tumor-specific antigens has emerged as a promising but challenging treatment option for malignant lymphomas. Follicular lymphoma is among the most common lymphomas worldwide and remains incurable for most patients. Considered to be an immunogenic disease it represents an interesting disease entity for various immunotherapeutic approaches.

In an article published in the May issue of *Clinical Cancer Research*, Nielsen and colleagues provided important proof-of-principle data on the immunogenicity of follicular lymphoma that might represent a first step towards personalized adoptive immunotherapies in this disease. The authors combined targeted next-generation sequencing and in silico analyses to explore the concept of somatic neoepitope prediction. Neoantigen-specific CD8^+^ T-cells could be identified in a small subset of patients selected for in vitro immunogenicity experiments, however at remarkably low frequencies and in only a few patients at single time-points. Of note, the immunogenic neoepitopes were derived from mutant *CREBBP* and *MEF2B*, two genes that have previously been shown to be functionally and prognostically relevant in this disease.

In this commentary we discuss the promises but also the challenges of how to translate these findings into clinical practice.

## Background

Harnessing the immune system to attack tumor cells has emerged as a promising but challenging treatment option for malignant lymphomas. A recent breakthrough in cancer immunotherapy has been declared when immune checkpoint blockade with antibodies directed against programmed-death 1 (PD-1) have resulted in objective response rates in up to 87% in patients with relapsed and refractory Hodgkin lymphomas [1, 2]. Non-Hodgkin lymphomas also respond to PD-1 blockade, including the two most common subtypes, follicular and diffuse large B-cell lymphomas, but the rate and quality of treatment responses are much less impressive [3].

In principle, PD-1 blockade acts by interfering with tumor-induced immune tolerance and unleashes a pre-exiting anti-tumor response directed against a variety of tumor-associated antigens, however also including epitopes that may not be tumor-specific and contribute to autoimmune-like or inflammatory side effects [4]. Also, low numbers and functionality of immune effector cells will limit the clinical efficacy of this approach.

Immune effector cells can be expanded ex vivo, an approach referred to as adoptive cellular immunotherapy. E.g., expanded autologous antitumor lymphocytes resulted in tumor regression in up to 70% in patients with melanoma [5]. Tumor-reactive T-cells have also been identified in lymphoid malignancies [6, 7].

Nielsen et al. recently provided interesting data that might represent a first step towards personalized adoptive immunotherapies in patients with follicular lymphoma [8].

## Main text

In their manuscript “Toward Personalized Lymphoma Immunotherapy: Identification of Common Driver Mutations Recognized by Patient CD8^+^ T Cells”, Nielsen et al. explored the concept of somatic neoepitope prediction and assessed the functionality of autologous CD8^+^ T-cells against tumor-specific antigens. To identify putative somatic neoepitopes, customized targeted next-generation sequencing was performed on 53 lymphoma samples, capturing ten genes that are known to be recurrently altered in malignant lymphomas and considered oncogenic drivers of the disease. Non-synonymous mutations were identified in 81% of patients. Using in silico algorithms, 37 of 43 patients harbored mutations that were predicted to form specific epitopes with sufficient binding affinity to the patients’ HLA class I haplotypes. From the 13 patients who were selected for in vitro immunogenicity experiments, three had detectable autologous mutation-specific CD8^+^ T-cells as confirmed by in vitro T-cell recognition of transfected autologous B-cells. The immunogenic neoepitopes were derived from mutant *CREBBP* and *MEF2B*, two genes that have previously been shown to be functionally and prognostically relevant in this disease [9–11].

### Conclusion and perspective

Can follicular lymphoma –again– serve as a prototype example for the successful introduction of innovative immunotherapeutic approaches? Two decades ago, the advent of monoclonal anti-CD20 antibodies marked the end of a treatment period now known as the pre-rituximab era. Generally considered an immunogenic disease with occasional waxing-and-waning lymphadenopathy and sporadic spontaneous regressions, follicular lymphomas can harbor more than 100 coding mutations that could potentially serve as tumor-specific neoepitopes [12]. Any mutation, including functionally irrelevant, so-called bystander mutations can produce immunogenic neoantigens, as long as they are transcribed and translated, and their gene products properly processed and presented onto a fitting HLA haplotype. An earlier study performed in melanoma patients receiving CTLA-4 antibodies could indeed demonstrate that the mutational load (and distinct neoantigen patterns) correlated with the immunogenicity and clinical benefit to immune checkpoint inhibition [13]. In that regard, it may come as a surprise that Nielsen et al. did not identify neoantigen-specific T-cells in the majority of patients with follicular lymphoma and that substantial efforts were required to detect some at remarkably low frequencies and in only a few patients at single time-points. On the other hand, it will be interesting to see if detectable neoantigen-reactive T-cells could serve as biomarkers to predict response to immune checkpoint inhibition in this disease.

It is likely that the authors would have identified more neoantigen-reactive T-cells in a higher fraction of patients with follicular lymphoma had they performed exome-wide analyses. However, the rationale behind targeting a limited number of gene mutations presumed to be acquired early in the molecular ontogeny of the disease and to drive the malignant phenotype is to minimize the risk of subclone selection and immune escape variants [14, 15]. Still, identifying these target genes remains a major challenge, given our incomplete understanding of the molecular biology of a disease as molecularly diverse and genetically unstable as follicular lymphoma. But even if directed against known driver gene mutations, immune evasion from effective CD8+ T-cell mediated anti-tumor responses might occur via loss of HLA, as recently described in a case of KRAS-mutant metastatic colorectal cancer [16].

Eventually, it remains to be proven if these autologous neoantigen-reactive CD8^+^ T-cells, even after ex vivo expansion, will elicit an effective immune response in patients and ultimately eradicate the disease. In contrast, engineered T-cells have already shown clinical activity. Promising response rates have been reported with autologous T-cells transduced with a chimeric antigen receptor directed against the pan B-cell marker CD19 for patients with refractory or relapsed B-cell malignancies [17]. To reduce on- and off-target toxicity, T-cells have been successfully engineered to target tumor-specific epitopes. E.g., engineered T-cells directed against the cancer-testis antigens NY-ESO-1 and LAGE-1 resulted in objective responses in 80% of patients with advanced multiple myeloma, without causing clinically apparent cytokine release syndromes [18].

In summary, from a scientific point of view, Nielsen et al. provide important proof-of-principle data on the immunogenicity of follicular lymphoma. From a translational research point of view, it remains unclear how to most effectively bring these findings into clinical practice. Rather exploratory, e.g. to determine the most promising neoantigen-haplotype patterns for immunotherapeutic approaches? Or diagnostically, e.g. as biomarkers to predict response to immune checkpoint inhibitors? Or therapeutically, e.g. as actual immune effector cells to personalize adoptive immunotherapy? From a clinical point of view, numerous questions remain to be addressed. E.g., how to select the subset of patients with follicular lymphoma who qualify for and are expected to gain most benefit from what type of personalized immunotherapy? How to incorporate personalized immunotherapeutic concepts into current treatment algorithms? And finally, how will they compare to the numerous other promising treatment options in terms of efficacy, toxicity, and –last but not least– cost? But for those of us who share Bon Scott’s Rock ‘n’ Roll point of view, all these challenges do not come as a surprise: It’s a long way to the top…

## References

[1] Ansell SM (2015). PD-1 blockade with nivolumab in relapsed or refractory Hodgkin’s lymphoma. N Engl J Med.

[2] Armand P, et al. Programmed death-1 blockade with pembrolizumab in patients with classical hodgkin lymphoma after brentuximab vedotin failure. J Clin Oncol. 2016;34(33):3733–39. 10.1200/JCO.2016.67.3467 PMC5791838 27354476

[3] Lesokhin AM (2016). Nivolumab in patients with relapsed or refractory hematologic malignancy: preliminary results of a phase Ib study. J Clin Oncol.

[4] Boussiotis VA (2016). Molecular and biochemical aspects of the PD-1 checkpoint pathway. N Engl J Med.

[5] Dudley ME (2002). Cancer regression and autoimmunity in patients after clonal repopulation with antitumor lymphocytes. Science.

[6] Rajasagi M (2014). Systematic identification of personal tumor-specific neoantigens in chronic lymphocytic leukemia. Blood.

[7] Riva G (2011). BCR-ABL-specific cytotoxic T cells in the bone marrow of patients with Ph(+) acute lymphoblastic leukemia during second-generation tyrosine-kinase inhibitor therapy. Blood Cancer J.

[8] Nielsen JS (2016). Toward personalized lymphoma immunotherapy: identification of common driver mutations recognized by patient CD8+ T cells. Clin Cancer Res.

[9] Pasqualucci L (2011). Inactivating mutations of acetyltransferase genes in B-cell lymphoma. Nature.

[10] Ying CY (2013). MEF2B mutations lead to deregulated expression of the oncogene BCL6 in diffuse large B cell lymphoma. Nat Immunol.

[11] Pastore A (2015). Integration of gene mutations in risk prognostication for patients receiving first-line immunochemotherapy for follicular lymphoma: a retrospective analysis of a prospective clinical trial and validation in a population-based registry. Lancet Oncol.

[12] Morin RD (2011). Frequent mutation of histone-modifying genes in non-Hodgkin lymphoma. Nature.

[13] Snyder A (2014). Genetic basis for clinical response to CTLA-4 blockade in melanoma. N Engl J Med.

[14] Kasajima A (2010). Down-regulation of the antigen processing machinery is linked to a loss of inflammatory response in colorectal cancer. Hum Pathol.

[15] Schreiber RD, Old LJ, Smyth MJ (2011). Cancer immunoediting: integrating immunity’s roles in cancer suppression and promotion. Science.

[16] Tran E, et al. T-Cell Transfer Therapy Targeting Mutant KRAS in Cancer. N Engl J Med. 2016;375(23):2255–62. 10.1056/NEJMoa1609279 PMC5178827 27959684

[17] Maude SL (2014). Chimeric antigen receptor T cells for sustained remissions in leukemia. N Engl J Med.

[18] Rapoport AP (2015). NY-ESO-1-specific TCR-engineered T cells mediate sustained antigen-specific antitumor effects in myeloma. Nat Med.

